# Testing for Drug-Related Infectious Diseases and Determinants among People Who Use Drugs in a Low-Resource Setting: A Respondent-Driven Cross-Sectional Survey

**DOI:** 10.3390/tropicalmed7090213

**Published:** 2022-08-29

**Authors:** Samuel Lazarus Likindikoki, Elia J. Mmbaga, Mucho Mizinduko, Mwijage Alexander, Lisa V. Adams, Robert Horsburgh, Kåre Moen, Germana Leyna, Theis Lange, Britt P. Tersbøl, Melkizedeck Leshabari, Dan W. Meyrowitsch

**Affiliations:** 1 Department of Epidemiology and Biostatistics, Muhimbili University of Health and Allied Sciences, Dar es Salaam 11101, Tanzania; 2 Department of Psychiatry and Mental Health, Muhimbili University of Health and Allied Sciences, Dar es Salaam 11101, Tanzania; 3 Department of Community Medicine and Global Health, Institute of Health and Society, University of Oslo, NO-0316 Oslo, Norway; 4 Department of Behavioural Sciences, Muhimbili University of Health and Allied Sciences, Dar es Salaam 11101, Tanzania; 5 Audrey and Theodor Geisel School of Medicine at Dartmouth, Hanover, NH 30001, USA; 6 Schools of Public Health, Boston University, Boston, MA 02108, USA; 7 Tanzania Food and Nutrition Centre, Dar es Salaam 11101, Tanzania; 8 Global Health Section, Department of Public Health, University of Copenhagen, 0917 Copenhagen, Denmark

**Keywords:** infectious diseases, HIV, TB, STIs, viral hepatitis, testing, PWUD, low-resource setting, Tanzania

## Abstract

(1) Background: There is a dearth of data on the levels and determinants of testing for drug-related infectious diseases among people who use drugs (PWUD). We assessed the proportions and determinants of testing for drug-related infectious diseases to inform ongoing interventions for PWUD. (2) Methods: A cross-sectional study involving 599 PWUD was conducted in Dar es Salaam and Tanga between January and February 2019. Data were collected through a researcher-administered questionnaire using handheld tablets. Logistic regression models were used to identify independent testing determinants for drug-related infectious diseases. (3) Results: A majority (98.0%) of participants were males, with a mean age of 36.8 (SD = 7.8) years. 75.0%, 40.6%, 38.6%, and 8.2% reported having ever tested for HIV, tuberculosis (TB), sexually transmitted infections (STIs), and viral hepatitis, respectively. The likelihood of HIV testing was higher among those living with someone (AOR = 2.18, 95% CI: 1.09–4.68) compared with those who were homeless and perceived treatment was appropriate (AOR = 2.18, 95% CI: 1.05–4.46), but was lower among those who experienced mild to moderate (AOR = 0.44, 95% CI: 0.21–0.95) and severe internalized stigma (AOR = 0.44, 95% CI: 0.22–0.94) compared with those reporting no internalized stigma, and among those who experienced financial difficulties resulting from spending on health care services (AOR = 0.60, 95% CI: 0.40–0.89). Perception of treatment appropriateness (AOR = 2.29, 96% CI: 1.10–5.06) and severe enacted stigma (AOR = 1.90, 95% CI: 1.06–3.42) were associated with increased odds of TB testing. The odds of STIs testing increased among those who were married (AOR = 2.31, 95% CI: 1.45–3.72) compared with those who were single and those who had experienced mild (AOR = 2.39, 95% CI: 1.28–4.53) or severe (AOR = 6.20, 95% CI: 1.99–23.83) sexual violence, compared with those who had not experienced sexual violence. However, the odds decreased among those who had been remanded in the past month (AOR = 0.64, 95% CI: 0.43–0.95) compared with those who were not remanded and among those who had financial difficulties resulting from spending on health care services (AOR = 0.66, 95% CI: 0.47–0.94). The likelihood of testing for viral hepatitis testing increased among those who had heard about the comprehensive HIV intervention package (CHIP) (AOR = 2.59, 95% CI: 1.40–4.94); however, it decreased among those who had financial difficulties resulting from spending on health care services (AOR = 0.48, 95% CI: 0.24–0.92). (4) Conclusions: Except for HIV, PWUD had undergone limited testing for drug-related infectious diseases. The study findings highlight some factors influencing testing for the selected infectious diseases investigated, which should be targeted for tailored interventions to improve diagnosis and treatment.

## 1. Introduction

Drug use is a significant problem globally. In 2018, 5.4% of the global population aged between 15 and 64 had used drugs at least once in the previous year, of whom 8.9% were in Africa [1,2]. About 13% of drug users suffer from drug use disorders, and less than 10% of them receive treatment [1]. Like many other countries, Tanzania is affected by the drug use problem. There are no country-level data to estimate the number of people who use drugs (PWUD) in Tanzania. However, in 2014, it was estimated that there were between 41,000–71,000 people who inject drugs (PWID) in Tanzania [3].

PWUD have increased morbidity and mortality risks partly due to comorbid health conditions [4,5]. HIV and other infectious diseases, including viral hepatitis, tuberculosis, and sexually transmitted infections (STIs), are commonly associated with drug use. About 62% of new adult HIV infections are among members of key populations and their partners, and 10% of new HIV infections are attributed to injection drug use [6]. The key populations are people at heightened risk of HIV due to higher-risk behaviors. HIV prevalence among PWUD was reported to be higher in eastern and southern African countries than in the general population [7]. As well as HIV, tuberculosis is among the comorbid conditions affecting PWUD. Studies have shown that a higher rate of tuberculosis is associated with comorbid HIV infection, homelessness, and drug use practices [8,9]. Tanzania is included among the countries with a high tuberculosis burden [10], and the burden is even higher for PWUD [11,12]. The global plan to end tuberculosis set an operational target of reaching 90% of people with tuberculosis who use drugs by improving access to services, screening, and active case finding, and then offering effective and affordable intervention [13]. Viral hepatitis is one of the infectious conditions affecting PWUD, and can be transmitted by sharing needles and syringes, use of unsterile equipment, and blood-blood contact. Viral hepatitis is prevalent among PWUD globally [14] and in Tanzania [8,15]. Viral hepatitis can cause serious health consequences including liver malfunction and cancer [16,17]. Moreover, sexually transmitted infections (STIs) are the other co-comorbid conditions affecting PWUD in high-income countries (HIC) [18] as well as low- and middle-income countries (LMIC) [19]. The health sector’s response to STIs has been cited by the World Health Organization (WHO) as critical to achieving universal health coverage [20].

Given the burden of these drug-related infectious diseases, testing is among the core elements of comprehensive HIV intervention for key and vulnerable populations. Testing allows early detection and treatment, decreasing levels of morbidity and mortality associated with comorbid infectious diseases. Realizing the need to address drug-related infectious diseases, the WHO included testing and treating comorbid conditions in the consolidated guidelines for HIV prevention, diagnosis, treatment, and care of key populations [21,22]. In addition to the WHO, the US Centre for Disease Control and Prevention (CDC) recommends integrated services for infectious diseases affecting PWUD [23]. In Tanzania, the national guidelines for a comprehensive package of HIV interventions for key populations has provided guidance on the integrated provision of health services for PWUD. The guidelines include, among other things, the need to address multiple comorbid conditions [24]. In addition, the guidelines address infectious diseases by including provisions related to HIV testing and counseling, presumptive STI screening, sexual and reproductive health (SRH), and screening for tuberculosis and viral hepatitis [24].

This notwithstanding, inadequate data are available to show the extent and correlations of testing for drug-related infectious diseases. The evidence indicates that testing for infectious diseases among PWUD is relatively low in certain areas [25,26,27,28,29] but higher in others [30,31]. Generally, data regarding access to testing for drug-related infectious diseases among PWUD in most African countries are lacking. Understanding the level of testing and associated factors among PWUD could help policy-makers, programmers, and non-governmental organizations to tailor drug-related infectious disease prevention and responses in this subpopulation. We used baseline data from a quasi-experimental study of a large key population [32] to understand the extent and determinants of testing for drug-related infectious diseases among PWUD. The specific objectives of this study were: (1) To determine the proportion of testing for selected drug-related infectious diseases among PWUD; (2) to examine individual and socio-structural factors associated with testing for drug-related infectious among PWUD.

## 2. Materials and Methods

The present study was part of a quasi-experimental study of a large key population [32]. Between January and February 2019, a cross-sectional baseline survey was conducted in Dar es Salaam and Tanga cities in the coastal area of Tanzania. Dar es Salaam is the largest commercial city in Tanzania, and Tanga is located 338 km north of Dar es Salaam. In 2020, Dar es Salaam and Tanga cities had estimated 5.5 million and 2.5 million populations, respectively [33]. Both cities are located on the coast of the Indian Ocean. According to the United Nations Office on Drugs and Crime (UNODC) report, coastal cities in East Africa have been transit points for drug trafficking from Europe, the Americas, and the Middle East [34]. Both cities are reported to have a relatively high number of PWUD [35]. The level of services for PWUD increased following the introduction of national guidelines for a comprehensive package of HIV interventions in 2012 [36]. In addition, methadone-assisted therapy (MAT) services were introduced in Tanga city a year after the baseline survey of the present study was conducted.

The study population comprised people who reported using drugs in the past year and lived in Dar es Salaam or Tanga. We included people who used drugs through injection and non-injection routes because both groups are at risk of adverse health consequences. Eligibility screening was conducted by screeners who themselves used drugs in the past. Eligibility criteria included having a valid study coupon, age 15 years or older, use of drugs through injection or non-injection routes in the past year, and reported to have been living in one of the study sites, i.e., Dar es Salaam or Tanga in the three months prior to the survey. The study used researcher-administered questionnaires on handheld tablets with an open data kit (ODK) application for data collection. Respondent-driven sampling (RDS) was used to recruit participants from the population of PWUD. RDS is an effective recruitment and sampling approach for hidden and hard-to-reach populations such as PWUD, where there is no sampling frame [37]. Five seeds from each site were recruited to support the initial stages of the recruitment process. Seeds were selected based on their diverse backgrounds to represent young and old, male and female, and those residing in different geographical locations within Dar es Salaam and Tanga cities. Each seed was given a maximum of three uniquely coded RDS coupons that were used to recruit up to three potential participants. Each recruited participant received coupons to recruit up to three other participants, and the process continued until the sample size was reached. The questionnaire was developed in English and translated into Kiswahili. The interviews were conducted using Kiswahili, an official language widely spoken in Tanzania. Data collection was completed through researcher-administered questionnaires using handheld tablets. Participants’ sociodemographic characteristics, self-reported information on testing for HIV, TB, STIs, and viral hepatitis, as well as socio-structural factors, were collected. Sociodemographic data (age, sex, education status, income level, homelessness status, and marital status) were obtained. In addition, the questionnaire captured access correlates such as the number of social supporters, how friendly or supportive healthcare providers were, perceived financial difficulties resulting from spending on healthcare, and experience of violence, stigma, and incarceration.

This study did not validate any tool, but the selected tools have been either previously used or validated in the local context. A conflict tactic scale (CTS) with 27 items was used to measure the experience of physical and sexual violence [38]. A total of ten and six items in the CTS were used to measure physical and sexual violence, respectively. Participants were told to state how often they had experienced certain things in the past, and the possible responses were: this never happened; happened one time; happened two to three times; happened four to ten times; or happened more than ten times. Social support was measured using a questionnaire with six items, SSQ-6 [39]. Participants indicated that up to nine people were available to provide support in each of the six domains included in the tool. Finally, the participants were asked to rate the overall level of satisfaction with support received from each of those mentioned supporters. A six-point Likert scale was used to indicate participants’ degree of satisfaction with the support from the people mentioned, ranging from “1—very dissatisfied” to “6—very satisfied”. The analysis contained in this paper used the reported number of social supporters as a measure to quantify the level of support.

The substance-use stigma mechanism scale (SU-SMS) was used to measure experienced and enacted stigma [40]. Internalized and enacted stigma subscales had a total of six items each. SU-SMS consisted of 12 items (six items for internalized stigma and six items for enacted stigma) scored on a five-point Likert scale. Participants’ responses ranged from “1—least stigmatizing” to “5—most stigmatizing”). Legal status: Apprehension (categorical, have you ever been apprehended, Yes/No); Sentenced (categorical, have you ever been sentenced, Yes/No); Jailed/remanded in the past 30 days (categorical, were you remanded or imprisoned in the last 30 days, Yes/No). Structural factors: Treatment was appropriate for the problem they had (Categorical, Yes/No); Experienced friendly health care last time they visited the health facility (Categorical, Yes/No); Experienced supportive health care providers last time they visited the health facility (Categorical, Yes/No); Heard about the existence of a comprehensive HIV intervention package for PWUD (CHIP) (Categorical, Yes/No); Perceived delays at health care facility (Categorical, Yes/No); Ever experienced financial difficulties as a result of spending on health care (Categorical, Yes/No). Statistical analyses were conducted using R-Studio, Version 1.2.1335 © 2009–2019 Inc.

The objective of the present study was to determine the proportion of drug-related infectious diseases ever tested for, i.e., HIV, tuberculosis, STIs, and viral hepatitis. Responses were dichotomized as “Yes” or “No”. Demographic data, the proportion of drug-related infectious diseases ever tested for, and proportions of predictors of interest were presented using descriptive statistics. Continuous variables were summarized using mean and corresponding standard deviation (SD) for normally distributed variables, and multivariable analyses were carried out to determine the association between determinants of testing for HIV, STIs, viral hepatitis, and tuberculosis. The mean and proportion differences between the sites were analyzed using chi-square tests. Unadjusted odds ratios (UOD) and adjusted odds ratios (AOR) with corresponding 95% confidence intervals (95% CI) were employed to report the associations between testing for drug-related infectious diseases and determinants of interest. The logistic regression models included variables with a *p*-value of <0.25 in bivariate analyses in order to minimize overfitting in the models. A *p*-value of <0.05 was considered statistically significant. RDS weighting was included for descriptive statistics but not for bivariate and multivariate analyses.

## 3. Results

We present our results on the proportions and determinants of testing for drug-related infectious diseases among people who use drugs in two coastal cities of Tanzania.

### 3.1. Participants’ Sociodemographic Characteristics

Table 1 summarizes the sociodemographic characteristics of the study participants. A total of 611 PWUD were recruited and participated in the study. Twelve participants did not complete the survey; therefore, only 599 participants were included in the final analysis. A majority of the participants were male (97.7%). About three-quarters of the participants were in the age range of 25 to 44 years. The overall mean age was 36.8 years (SD = 7.8), and the mean age was significantly higher among participants in Tanga at 38.0 years (SD = 8.02) than in Dar es Salaam at 35.61 years (SD = 7.49), (*p* < 0.05). About three-quarters of participants reported having attended or completed a primary level of education. Slightly more than half of the participants reported a monthly income of 200,000 Tanzanian Shillings (Tshs) {the equivalent of 87 USD or less}. About four out of ten participants reported being single, and the proportion of those who reported being single was significantly higher in Dar es Salaam than in Tanga (*p* < 0.05). About one out of ten participants reported being homeless. The proportion of participants who reported being homeless was significantly higher in Dar es Salaam than in Tanga (*p* < 0.05).

### 3.2. Proportion of Social Structural Determinants

About four out of ten participants reported having experienced physical violence. The mean score for physical violence was 12.05 (SD = 9.7, range = 0–45). About one out of ten participants reported having experienced a severe form of physical violence. Approximately one out of ten participants reported having ever experienced sexual violence. The mean score for sexual violence was 2.3 (SD = 3.65, range = 0–24). Most participants (82.3%) reported having experienced enacted stigma. Overall, the mean score for enacted stigma was 13.7 (SD = 6.0, range 0–30). The proportion of participants who reported having experienced a severe form of enacted stigma was higher in Dar es Salaam than in Tanga (*p* < 0.05). Most participants (93.97%) were found to have internalized stigma. Overall, the mean score for internalized stigma was 19.2 (SD = 7.02, range 0–24). Most participants (95.5%) reported having ever been apprehended, four out of ten reported being sentenced, and seven out of ten reported being jailed or remanded in the past month. While the proportion of participants who reported having ever been sentenced was significantly higher in Dar es Salaam than in Tanga (*p* < 0.05), the proportion of participants who reported being jailed or remanded in the past month was lower in Dar es Salaam than in Tanga (*p* < 0.05).

A majority of participants reported perceiving that when they last visited a health facility for care, the health care providers were friendly (88.9%) and supportive (89.8%) and that treatment was appropriate for the problem they had (89.5). There were no statistically significant differences between Dar es Salaam and Tanga regarding these perceptions.

About six out of ten participants reported having two or more social supporters. The mean score for the number of social supporters was 2.0 (SD = 1.51, range 0–9). The proportion of participants with two or more social supporters was significantly higher in Tanga than in Dar es Salaam (*p* < 0.05). About half of the participants reported having experienced financial difficulties due to spending on healthcare services. Between Dar es Salaam and Tanga, there was no statistically significant difference regarding having ever experienced financial difficulties due to spending on healthcare services (Table 1).

### 3.3. Proportions of Participants Who Tested for Drug-Related Infectious Diseases

Table 2 summarizes the proportion of testing for drug-related infectious diseases among study participants by study site. Of 599 participants included in our analysis, the proportion tested for HIV was 75.0% [95% CI, 71.5–78.4], tested for TB was 40.57% [95% CI, 36.7–44.6], tested for STIs was 38.56% [95% CI, 34.8–42.5] and tested for viral hepatitis was 8.18% [95% CI, 6.2–10.7]. The proportions of participants tested for TB and viral hepatitis were significantly higher among participants in Dar es Salaam than in Tanga (*p* < 0.05). In contrast, there were no statistically significant differences regarding testing for HIV and STI.

### 3.4. Access to Drug-Related Infectious Diseases Testing among People Who Use Drugs

Table 3 summarizes the results of the bivariate analyses, indicating that testing for HIV was associated with the level of income, homelessness, criminalization [apprehension, ever sentenced, jailed or remanded in the past month], perception of appropriate treatment at a health facility, perception of health care providers as supportive and friendly, and ever having experienced financial difficulties as a result of spending on healthcare (*p* < 0.05). TB testing was associated with severe enacted stigma, having been jailed or remanded in the past month, perception of appropriate treatment at a health facility, perception of supportive health care providers, and reporting ever having experienced financial difficulties due to spending on healthcare (*p* < 0.05). STI testing was associated with marital status, sexual violence, ever being sentenced, being jailed or remanded in the past month, perception of appropriate treatment at a health facility, and reporting ever having experienced financial difficulties as a result of spending on healthcare (*p* < 0.05). Lastly, testing for viral hepatitis was associated with marital status, experiencing physical violence, and having heard about the existence of a comprehensive package for HIV intervention for people who use drugs (*p* < 0.05).

### 3.5. Determinants of Testing for Drug-Related Infectious Diseases

Determinants of testing for HIV, TB, STIs, and viral hepatitis are summarized in Table 4. Overall, financial difficulties resulting from spending on health care internalized and enacted stigma, sexual violence, criminalization, and perception of the appropriateness of services predicted access to one or more testing for infectious diseases. Multivariable logistic regression analyses were done to estimate independent determinants of testing for HIV, TB, STIs, and viral hepatitis. After adjusting for confounding factors, testing for HIV increased among participants who reported to have been living together with someone (AOR = 2.8, 95% CI: 1.09–4.68) as compared to those who were homeless and among those who perceived the treatment at a health facility was appropriate for their needs (AOR = 2.18, 95% CI: 1.05–4.46). However, the odds of testing for HIV decreased among participants who reported having; experienced financial difficulties as a result of spending on health care (AOR = 0.60, 95% CI: 0.40–0.89), mild to moderate internalized stigma (AOR = 0.44, 95% CI: 0.21–0.95), and severe internalized stigma (AOR = 0.44, 95% CI: 0.22–0.94) compared to those who reported not to experience internalized stigma. The odds of testing for TB increased among those who perceived that the treatment at the health facility was appropriate for their need (AOR = 2.29, 95% CI: 1.10–5.06) and had experienced severe enacted stigma (AOR = 1.90, 95% CI: 1.06–3.42) as compared to those who did not experience enacted stigma. The odds of testing for STIs increased among married (AOR = 2.31, 95% CI: 1.45–3.72) compared to single participants, among those who reported having experienced mild (AOR = 2.39, 95% CI: 1.28–4.53) and severe (AOR = 6.20, 95% CI: 1.99–23.83) sexual violence as compared to those who have not experienced sexual violence and those who perceived the treatment at the health facility was appropriate (AOR = 2.24, 95% CI: 1.03–5.11). However, the odds of testing for STIs decreased among those who reported having been jailed or remanded in the past 30 days (AOR = 0.64, 95% CI: 0.43–0.95) compared to those who were not remanded and have experienced financial difficulties due to healthcare spending (AOR = 0.66, 95% CI: 0.47–0.94) than those who did not experience financial difficulties. The odds of testing for viral hepatitis increased among participants who reported having heard about a comprehensive HIV intervention package for PWUD (CHIP) (AOR = 2.59, 95% CI: 1.40–4.94) than those who did not hear about CHIP but decreased among those who reported having experienced financial difficulties as a result of spending on health care services (AOR = 0.48, 95% CI: 0.24–0.92) than those who did not experience financial difficulties.

## 4. Discussion

The main aim of this study was to ascertain the proportions of people who use drugs who had been tested for drug-related infectious diseases. The drug-related infectious diseases testing studied included HIV, TB, STIs, and viral hepatitis. Moreover, the study was set to determine the independent correlates of testing for the selected drug-related infectious diseases among people who use drugs. Contrary to our hypothesis, the results showed that the proportion who had undergone testing for HIV was relatively modest, and was more limited for TB, STIs, and viral hepatitis. Perceived financial difficulties resulting from spending on health care services, perception about appropriate treatment for the needs of PWUD at health care facilities, stigma, sexual violence, and criminalization influenced access to testing for one or more drug-related infectious diseases. Our study found that the proportions of study subjects tested for TB and viral hepatitis were significantly higher in Dar es Salaam than in Tanga. However, there was no difference between Dar es Salaam and Tanga in the proportions who had been tested for HIV and STIs.

The observed differences might be because HIV and STI services are integrated within the primary health care system and hence universally accessible by all subpopulations, including PWUD. However, testing for tuberculosis and viral hepatitis run parallel to the mainstream health care system and are uniquely tailored to vulnerable populations such as PWUD. Testing for tuberculosis and viral hepatitis can easily be accessed through MAT clinics, because the tests are offered as part of the integrated services. Dar es Salaam city started to offer MAT services in 2012. At the time the present study was conducted, there were about three MAT clinics in Dar es Salaam and none in Tanga.

Our study found that three-quarters of the sampled population had ever been tested for HIV. While this testing proportion seems relatively high compared to testing for other drug-related infections in the present study, it falls short of the UNAIDS global target of having 95% of people tested for HIV by 2030 [10]. Other studies in Russia, the USA, and Cambodia have found similar results [25,41,42]. Contrary to the present studies, research undertaken in Dar es Salaam about eight years ago found that only about a third (36.0%) of the prospective cohort of people who injected drugs had been tested for HIV [29]. The present reported level of HIV testing may indicate that an increasing proportion of drug users are opting for HIV testing. Given the high prevalence of HIV among people who use drugs [43,44] and that 10% of new HIV infections are due to drug use [6], it is imperative to step up HIV testing for PWUD as one of the key HIV prevention strategies.

Despite the higher prevalence of TB among people who use drugs in Tanzania [11], the present study found that only four out of ten participants reported having ever been tested for TB. In contrast to our findings, a US study reported that 95% of PWUD had ever been tested for TB [45]. There is limited literature available on the levels of testing for tuberculosis among people who use drugs. The available literature focuses on the prevalence and uptake of tuberculosis treatment, but not on testing. Therefore, it is unclear what are the contextual contributing factors to the observed low rate of tuberculosis testing. Further qualitative studies will be instrumental in providing insight in this regard. Prioritizing TB testing among high-risk groups such as people who use drugs is one of the essential strategies to increase case notification and treatment, and reduce TB-related morbidity and mortality. Therefore, it is essential to use an equitable, rights-based, people-centered approach to increase tuberculosis screening among PWUD.

We found a relatively low proportion of testing for STIs among the study participants, with only 38.6% reporting ever having been tested for STIs. Furthermore, we found that married participants were likelier to have been tested for STIs. However, being incarcerated negatively influenced STI testing. Increased STI testing uptake among couples is likely to be due to the current practice that encourages partner notification, as it is indicated in the national STI guidelines that partner notification tends to break the chain of STIs transmission [46]. Also, STIs have immediate and observable symptoms, so if one of the partners gets infected, it can generate discussion among partners and facilitate the perceived need for a test and treatment. In addition, incarcerated people who use drugs might have limited access to general health services, including STI screening. Information on the extent of STI screening among people who use drugs is scarce. Generally, STIs are comorbid conditions among people who use drugs, due to blood-borne exposure and risky sexual behaviors. Therefore, primary clinical and laboratory screening for STIs is critical and is likely to reduce the burden associated with co-morbidity [46].

Alarmingly, a tiny proportion of the study participants reported having ever been tested for viral hepatitis. This indicates structural issues for such services in Tanzania. Similar to our findings, a study in Vietnam reported that approximately a third of people who inject drugs reported ever being tested for hepatitis C [47]. Studies in Tanzania have indicated a high burden of viral hepatitis among people who use drugs. Therefore, there is an urgent need to routinize testing for hepatitis B surface antigen (HBsAg) and hepatitis C virus [2,15]. We found that living with someone, compared with being homeless, decreased the odds of testing for HIV but not TB, STIs, or viral hepatitis. It is unclear why living with someone influenced testing for HIV. However, it might be because of the potential material and or emotional support provided by the people living with them, given other socio-cognitive factors such as perceived HIV-related stigma.

Experience of sexual violence increased the odds of testing for STIs, but not HIV, tuberculosis, or viral hepatitis. It is unclear why sexual violence influences such access to STI testing. One possible explanation could be that people exposed to sexual violence also engage in a higher level of STI-related risky behavior. Therefore, they are more motivated to test for STIs since they are at higher risk of STIs. Sexual violence is thereby an indirect proxy for a higher risk of STIs. Further qualitative research is warranted to understand better the context in which the experience of sexual violence influences testing for STIs.

Stigma was one of the correlates associated with testing for TB and HIV. While enacted stigma increased the odds of testing for tuberculosis, internalized stigma negatively influenced testing for HIV. It is plausible that PWUD with overt tuberculosis symptoms might be stigmatized due to their appearance, and that situation might heighten their need to access testing and treatment. On the other hand, felt stigma might make PWUD more vulnerable and decrease their perceived need to access testing for HIV.

We found that the perception that treatment at health care facilities was appropriate for the needs of PWUD increased the odds of testing for HIV, tuberculosis, and STIs. The acceptability of services rendered partly explains the reason for the utilization of services. This might be because participants did not perceive the presence of certain structural factors, such as having to spend a long time at a health facility, that could have competed with engagement in other gainful activities. It can also be explained by the perception that there are favorable arrangements at the health facilities, such as opioid substitution therapy clinics, that could meet the unique needs of people who inject drugs.

Meta-analysis has indicated that opioid substitution therapy may increase access to HIV testing among people who inject drugs [48]. Experience of financial difficulties as the result of spending on health care services negatively influenced testing for HIV, tuberculosis, and viral hepatitis. Income is a crucial element that can facilitate the use of health services. Similarly, a study in the USA reported that financial burden was among the challenges in accessing care faced by individuals using illicit drugs [49].

The present study has several strengths and limitations which should be considered when interpreting the results. First, our study assessed several drug-related infectious diseases simultaneously, while other previous studies have looked at one or two infectious diseases. Second, our study used a larger sample of people who use drugs from within the communities of two major cities with a high burden of infections. Third, we considered arrays of factors influencing access to drug-related infectious diseases, which could help shape interventions. However, testing was self-reported and may have introduced recall and social desirability biases. Secondly, we asked participants to report if they had ever been tested for these infectious diseases, which limited our ability to understand recent behaviors and might have introduced recall bias. Thirdly, while RDS is effective at reaching hidden and hard-to-reach populations such as PWUD, it is prone to selection bias.

## 5. Conclusions

This study has indicated the proportions, correlation, and implications for ongoing HIV prevention intervention to address comorbid conditions among PWUD. Generally, we found a modest proportion of PWUD had been tested for HIV, and meager proportions reported having been tested for TB, STIs, and viral hepatitis. Perceived financial difficulties as the result of spending on health care services, perceptions of receiving treatment appropriate to the needs of PWUD, stigma, sexual violence, and criminalization correlated with access to testing for one or more drug-related infectious diseases. From these findings, ongoing comprehensive HIV intervention should factor in social enablers such as health care expenditure, stigma, violence, and criminalization, and have appropriate treatment tailored to address the unique needs of people who use drugs.

## Figures and Tables

**Table 1:** Demographic, social, and structural characteristics of the study participants in Dar es Salaam and Tanga (*n* = 599).

Characteristics	Overall *n* = 599		Dar es Salaam *n* = 311		Tanga *n* = 288		# *p*-Value
	*n* (%) ‡	% †	*n* (%) ‡	% †	*n* (%) ‡	% †	
Age (years)	36.76 (7.84)		35.61 (7.49)		38.00 (8.02)		<0.001 **
Age categories							0.028 *
<25	40 (6.68)	(6.34)	26 (8.36)	7.89	14 (4.86)	4.99	
25–34	258 (43.07)	(43.95)	146 (46.95)	49.67	112 (38.89)	39.03	
35–44	216 (36.06)	(34.58)	101 (32.48)	30.94	115 (39.93)	37.72	
45+	85 (14.19)	(15.13)	38 (12.22)	11.49	47 (16.32)	18.26	
Sex							0.875
Male	587 (98.00)	(97.69)	304 (97.75)	97.33	283 (98.26)	97.99	
Female	12 (2.00)	(2.31)	7 (2.25)	2.27	5 (1.74)	2.01	
Education status							0.878
None	27 (4.51)	(5.13)	15 (4.82)	5.64	12 (4.17)	4.68	
Primary	451 (75.29)	(73.97)	235 (75.56)	73.63	216 (75.00)	74.27	
Secondary+	121 (20.20)	(20.90)	61 (19.62)	20.73	60 (20.83)	21.05	
Income status (Tshs)							0.181
<50,000	153 (25.54)	(26.43)	69 (22.19)	19.83	84 (29.17)	32.12	
50,001–120,000	59 (9.85)	(9.15)	29 (9.32)	9.01	30 (10.42)	9.27	
120,001–200,000	108 (18.03)	(17.35)	62 (19.94)	20.87	46 (15.97)	14.32	
>200,000	279 (46.58)	(47.07)	151 (48.55)	50.30	128 (44.44)	44.29	
Marital status							
Single	263 (43.91)	(43.60)	163 (52.41)	52.80	67 (34.70)	35.69	<0.001 **
Married	125 (20.87)	(20.76)	58 (18.65)	18.90	100 (23.30)	22.36	
Separated/divorced	211 (35.2)	(35.6)	90 (28.9)	28.3	121 (42.0)	42.0	
Residence Status							<0.001 **
Homeless	64 (10.7)	(9.6)	49 (15.8)	14.9	15 (5.2)	5.0	
Living with someone	295 (49.3)	(49.4)	154 (49.5)	50.2	141 (49.0)	48.7	
Rent/own a house	240 (40.0)	(41.0)	108 (34.7)	34.9	132 (45.8)	46.3	
Physical violence							
Mean (SD), range = 0–45	12.1 (9.7)		12.0 (9.7)		12.2 (9.8)		0.784
None	353 (58.9)	(58.7)	186 (59.8)	58.91	167 (58.0)	58.54	0.818
Mild only	178 (29.7)	(30.3)	92 (29.6)	31.80	86 (29.9)	28.92	
Severe	68 (11.4)	(11.0)	33 (10.6)	9.30	35 (12.1)	12.54	
Sexual violence							
Mean (SD), range = 0–24	2.3 (3.7)		2.1 (3.5)		2.6 (3.8)		0.055
None	530 (88.5)	(88.9)	280 (90.0)	91.6	250 (86.8)	86.55	0.463
Mild only	53 (8.8)	(8.5)	24 (7.7)	6.7	29 (10.1)	10.01	
Severe	16 (2.7)	(2.6)	7 (2.3)	1.7	9 (3.1)	3.44	
Enacted stigma							
Mean (SD), range 0–30	13.7 (6.0)		14.0 (6.0)		13.3 (5.9)		0.117
None	106 (17.7)	(17.8)	52 (16.7)	16.3	54 (18.7)	19.2	0.036
Mild to moderate	380 (64.4)	(63.7)	188 (60.5)	59.5	192 (66.7)	67.2	
Severe	113 (18.9)	(18.5)	71 (22.8)	24.2	42 (14.6)	13.6	
Internalized stigma							
Mean (SD), range 0–24	19.2 (7.0)		18.8 (7.2)		19.6 (6.8)		0.138
None	37 (6.2)	(6.0)	18 (5.8)	5.39	19 (6.6)	6.5	0.031
Mild to moderate	228 (38.1)	(39.5)	134 (43.1)	42.04	94 (32.6)	37.4	
Severe	334 (55.7)	(54.5)	159 (51.1)	5.39	175 (60.8)	56.1	
Ever apprehended							0.844
Yes	574 (95.83)	(95.5)	299 (96.1)	95.7	275 (95.5)	95.5	
No	25 (4.2)	(4.4)	12 (3.9)	4.3	13 (4.5)	4.5	
Ever sentenced							<0.001
Yes	243 (40.6)	(41.4)	100 (32.2)	29.7	143 (49.6)	51.4	
No	356 (59.4)	(58.6)	211 (67.8)	70.3	145 (50.4)	48.6	
Jailed/remanded past month							<0.001
Yes	414 (69.1)	(68.0)	237 (76.2)	76.0	177 (61.5)	61.2	
No	185 (30.9)	(32.0)	74 (23.3)	24.0	111 (38.5)	38.8	
Ever heard about CHIP							0.069
Yes	263 (43.9)	(45.9)	125 (40.2)	40.28	138 (47.92)	50.74	
No	336 (56.1)	(54.1)	186 (59.8)	59.72	150 (53.08)	49.26	
Appropriate treatment							0.098
Yes	536 (89.48)	(90.31)	285 (91.6)	92.1	251 (87.2)	88.8	
No	63 (10.52)	(9.69)	26 (8.4)	7.9	37 (12.8)	11.2	
HCW are friendly							0.976
Yes	527 (88.0)	(88.8)	273 (87.8)	87.8	254 (88.2)	89.7	
No	72 (12.0)	(11.2)	38 (12.2)	12.2	34 (11.8)	10.3	
HCW are supportive							0.823
Yes	538 (89.8)	(90.4)	278 (89.4)	88.5	260 (90.3)	92.1	
No	61 (10.2)	(9.6)	33 (10.6)	11.5	28 (9.7)	7.9	
Delays at the health facility							0.785
Yes	204 (34.1)	(32.2)	108 (34.7)	34.4	96 (33.3)	30.3	
No	395 (65.9)	(67.8)	203 (65.3)	65.6	192 (66.7)	69.7	
Financial difficulties							0.174
Yes	324 (54.1)	(55.0)	177 (56.9)	57.6	147 (51.0)	53.1	
No	275 (45.9)	(45.0)	134 (46.1)	42.4	141 (49.0)	46.9	
Social supporters							
One	245 (40.9)	(40.7)	139 (44.7)	43.9	106 (36.8)	38.0	<0.001
More than one	354 (59.1)	(59.3)	172 (55.3)	56.1	182 (63.2)	62.0	

Note: % ‡ = unweighted proportions, % † = weighted proportions, SD = standard deviation, HCW = health care workers, CHIP = comprehensive HIV intervention package, # *p* for chi-square test, * = *p* < 0.05, ** = *p* < 0.01, Tshs = Tanzanian Shillings.

**Table 2:** Proportions of people who use drugs who reported ever being tested for drug-related infections and diseases, stratified by study site, Dar es Salaam and Tanga, in 2019, (*n* = 599).

Variable	Overall *n* = 599	Study Site		*p*-Value *#*
		Dar es Salaam *n* = 311	Tanga *n* = 288	
	Yes (%)	Yes (%)	Yes (%)	
Reported tested for:				
HIV	450 (75.1)	243 (78.1)	207 (71.9)	0.094
TB	243 (40.6)	162 (52.1)	81 (28.1)	<0.001
STIs	231 (38.6)	127 (40.8)	104 (36.1)	0.270
Viral Hepatitis	49 (8.2)	37 (11.9)	12 (4.2)	0.001

Note: HIV = Human Immunodeficient Virus, TB = Tuberculosis, STIs = Sexually Transmitted Infections. Viral hepatitis includes hepatitis B and C. # = *p* for the chi-square test.

**Table 3:** Results of bivariate analyses of the associations between predictors and access to testing for drug-related infectious diseases among people who inject drugs in Dar es Salaam and Tanga (*n* = 599).

Predictors	HIV	TB	STIs	Hepatitis
	UOR (95% CI)	UOR (95% CI)	UOR (95% CI)	UOR (95% CI)
Age categories				
<25	Ref	Ref	Ref	Ref
25–34	0.67 (0.33–1.38)	1.00 (0.51–1.98)	1.61 (0.82–3.14)	1.80 (0.41–6.07)
35–44	0.73 (0.35–1.51)	1.01 (0.51–2.01)	1.39 (0.71–2.75)	1.03 (0.24–3.32)
45+	0.52 (0.22–1.21)	0.82 (0.38–1.77)	1.36 (0.64–2.89)	0.94 (0.19–3.65)
Education status				
None	Ref	Ref	Ref	Ref
Primary	0.82 (0.35–1.93)	0.44 (0.17–1.11)	0.56 (0.23–1.35)	1.12 (0.25–4.97)
Secondary+	0.62 (0.24–1.58)	0.30 (0.11–0.79) *	0.50 (0.19–1.26)	0.49 (0.11–2.26)
Income status (Tshs)				
<50,000	Ref	Ref	Ref	Ref
50,001–120,000	1.94 (0.99–3.77)	1.30 (0.69–2.42)	0.92 (0.49–1.69)	0.76 (0.25–2.31)
120,001–200,000	1.66 (0.95–2.93)	0.97 (0.59–1.59)	1.07 (0.64–1.77)	1.01 (0.37–2.74)
>200,000	1.13 (0.69–1.82)	1.07 (0.72–1.60)	0.99 (0.66–1.49)	0.65 (0.31–1.39)
Residence Status				
Homeless	Ref	Ref	Ref	Ref
Living with someone	2.08 (1.04–4.17) **	1.40 (0.81–2.41)	0.87 (0.49–1.53)	1.00 (0.37–2.74)
Rent/own a house	1.24 (0.60–2.54)	1.26 (0.72–2.19)	0.76 (0.43–1.35)	0.88 (0.32–2.44)
Marital status				
Single	Ref	Ref	Ref	Ref
Married	0.78 (0.47–1.31)	1.12 (0.72–1.72)	0.46 (0.29–0.71) **	0.57 (0.26–1.26)
Separated/divorced	1.10 (0.73–1.67)	1.09 (0.75–1.57)	0.90 (0.62–1.33)	0.52 (0.26–1.03)
Physical violence				
None	Ref	Ref	Ref	Ref
Mild only	0.93 (0.61–1.41)	1.13 (0.78–1.63)	0.91 (0.63–1.32)	1.45 (0.69–3.05)
Severe	0.67 (0.35–1.28)	0.72 (0.43–1.22)	0.75 (0.44–1.26)	0.45 (0.21–0.95) **
Sexual violence				
None	Ref	Ref	Ref	Ref
Mild only	1.34 (0.72–2.49)	1.49 (0.79–2.91)	0.46 (0.25–0.85) ***	1.08 (0.37–4.32)
Severe	1.04 (0.33–3.27)	0.91 (0.29–2.91)	0.19 (0.04–0.63) ***	0.62 (0.14–5.79)
Enacted stigma				
None	Ref	Ref	Ref	Ref
Mild to moderate	0.76 (0.47–1.23)	0.76 (0.49–1.19)	1.00 (0.64–1.57)	0.80 (0.36–1.79)
Severe	0.76 (0.42–1.39)	0.56 (0.33–0.97) *	0.82 (0.48–1.41)	1.46 (0.49–4.34)
Internalized stigma				
None	Ref	Ref	Ref	Ref
Mild to moderate	0.39 (0.19–0.82) *	0.65 (0.28–1.42)	1.08 (0.49–2.32)	0.67 (0.07–2.99)
Severe	0.41 (0.21–0.83) *	0.72 (0.32–1.54)	1.09 (0.51–2.31)	0.60 (0.07–2.26)
Ever apprehended				
No	Ref	Ref	Ref	Ref
Yes	0.48 (0.19–1.22)	0.82 (0.31–2.00)	0.61 (0.21–1.56)	2.45 (0.14–1.87)
Ever sentenced				
No	Ref	Ref	Ref	Ref
Yes	0.69 (0.48–1.03)	1.02 (0.73–1.42)	0.69 (0.50–0.98) **	1.19 (0.63–2.32)
Jailed/remanded past 30 days				
No	Ref	Ref	Ref	Ref
Yes	0.59 (0.40–0.87) **	0.69 (0.48–0.99) *	0.61 (0.43–0.89) ***	1.61 (0.88–2.93)
Ever heard about CHIP				
No	Ref	Ref	Ref	Ref
Yes	1.78 (0.81–1.71)	1.34 (0.96–1.86)	1.35 (0.97–1.88)	2.60 (1.41–4.79) *
Treatment is appropriate				
No	Ref	Ref	Ref	Ref
Yes	2.36 (1.37–4.05) **	2.61 (1.41–4.84) ***	2.80 (1.004–3.22) *	1.35 (0.47–3.89)
Providers are friendly				
No	Ref	Ref	Ref	Ref
Yes	1.86 (1.10–3.13) **	1.53 (0.90–2.58)	0.83 (0.49–1.39)	1.02 (0.42–2.49)
Providers are supportive				
No	Ref	Ref	Ref	Ref
Yes	1.98 (1.14–3.45) *	2.05 (1.13–3.72) **	1.57 (0.88–2.79)	1.30 (0.45–3.75)
Delays at the health facility				
No	Ref	Ref	Ref	Ref
Yes	1.01 (0.68–1.49)	0.99 (0.70–1.40)	0.82 (0.58–1.16)	1.07 (0.57–1.99)
Financial difficulties				
No	Ref	Ref	Ref	Ref
Yes	0.55 (0.39–0.80) ***	0.70 (0.51–0.98) **	0.61 (0.44–0.86) ***	0.44 (0.22–0.87) **
Social supporters				One
One	Ref	Ref	Ref	Ref
More than one	1.16 (0.79–1.69)	1.21 (0.87–1.68)	0.88 (0.63–1.23)	1.09 (0.60–1.97)

Note: Significance codes: ‘*’ *p* < 0.05, ‘**’ *p* < 0.01, ‘***’ *p* < 0.001, UOR = unadjusted odds ratio, CHIP = comprehensive HIV intervention package, CI = confidence interval, HIV = human immunodeficiency virus, TB = tuberculosis, STIs = sexually transmitted infections, Ref = reference group. Viral hepatitis included hepatitis B and C, Tshs = Tanzania Shillings.

**Table 4:** Multivariate analyses for predictors of testing for drug-related infectious diseases among people who use drugs in Dar es Salaam and Tanga, Tanzania 2019, (*n* = 599).

Predictors	HIV	TB	STIs	Viral Hepatitis
	AOR (95% CI)	AOR (95% CI)	AOR (95% CI)	AOR (95% CI)
Age				
<25	NA	NA	Ref	NA
25–34	NA	NA	0.51 (0.25–1.02)	NA
35–44	NA	NA	0.53 (0.26–1.10)	NA
45+	NA	NA	0.57 (0.25–1.29)	NA
Residence Status				
Homeless	Ref	NA	NA	NA
Living with someone	2.18 (1.09–4.68) *	NA	NA	NA
Rent/own a house	1.31 (0.64–2.91)	NA	NA	NA
Marital status				
Single	NA	NA	NA	Ref
Married	NA	NA	2.31 (1.45–3.72) ***	1.78 (0.89–3.67)
Separated/divorced	NA	NA	1.15 (0.75–1.74)	1.69 (0.74–3.82)
Physical violence				
None	Ref	Ref	NA	Ref
Mild only	0.99 (0.62–1.56)	0.81 (0.55–1.20)	NA	0.83 (0.36–1.77)
Severe	0.82 (0.39–1.64)	1.05 (0.59–1.87)	NA	2.16 (0.93–4.78)
Sexual violence				
None	NA	NA	Ref	NA
Mild only	NA	NA	2.39 (1.28–4.53) **	NA
Severe	NA	NA	6.20 (1.99–23.83) **	NA
Enacted stigma				
None	NA	Ref	NA	NA
Mild to moderate	NA	1.31 (0.83–2.10)	NA	NA
Severe	NA	1.90 (1.06–3.42) *	NA	NA
Internalized stigma				
None	Ref	NA	NA	NA
Mild to moderate	0.44 (0.21–0.95) *	NA	NA	NA
Severe	0.44 (0.22 -0.94) *	NA	NA	NA
Ever apprehended				
No	Ref	NA	NA	Ref
Yes	0.9 1 (0.36–2.39)	NA	NA	0.45 (0.14–1.8)
Ever sentenced				
No	Ref	NA	Ref	NA
Yes	0.73 (0.47–1.11)	NA	0.88 (0.61–1.28)	NA
Jailed/remanded past 30 days				
No	Ref	Ref	Ref	Ref
Yes	0.70 (0.45–1.09)	0.73 (0.50–1.06)	0.64 (0.43–0.95) *	0.65 (0.34–1.29)
Ever heard about CHIP				
No	NA	Ref	Ref	Ref
Yes	NA	1.26 (0.90–1.77)	1.29 (0.91–1.83)	2.59 (1.40–4.94) **
Treatment is appropriate				
No	Ref	Ref	Ref	NA
Yes	2.18 (1.05–4.46) *	2.29 (1.10–5.06) *	2.23 (1.03–5.07) *	NA
Providers are supportive				
No	Ref	Ref	Ref	NA
Yes	1.33 (0.62–2.78)	1.36 (0.65- 2.91)	1.25 (0.58–2.75)	NA
Financial difficulties				
No	Ref	Ref	Ref	Ref
Yes	0.60 (0.40–0.89) *	0.74 (0.53–1.05)	0.66 (0.47–0.94) *	0.48 (0.24–0.92) *

Note: Significance codes: ‘*’ *p* < 0.05, ‘**’ *p* < 0.01, ‘***’ *p* < 0.001, AOR = Adjusted Odds Ratio, CHIP = comprehensive HIV intervention package, CI = confidence Interval, HIV = Human Immunodeficient Virus, TB = Tuberculosis, STIs = Sexually Transmitted Infections, Ref = Reference Group, Viral hepatitis= Included hepatitis B and C, NA = Not Applicable.

## Data Availability

The full dataset is not publicly available due to privacy concerns because of the nature of the study. Data can be made available by reasonable request through the corresponding author.
